# Real-Time PCR Test (Flora Select™) for Assessing the Effectiveness of Bacterial Vaginosis Treatment During Pregnancy

**DOI:** 10.3390/microorganisms13092169

**Published:** 2025-09-17

**Authors:** Hajime Ota, Shigeki Shimada, Yuta Kobayashi, Tatsuya Yoshiwara, Osamu Yoshino, Yoshiyuki Fukushi, Shinichiro Wada, Soromon Kataoka, Hideto Yamada

**Affiliations:** 1 Department of Obstetrics and Gynecology, Sapporo Toho Hospital, Kita 17-jo, Higashi 15-chome, 3-1, Higashi-ku, Sapporo 065-0017, Hokkaido, Japan; hjm.ohta@gmail.com; 2 Department of Obstetrics and Gynecology, Teine Keijinkai Hospital, 1-40, 12-chome, Maeda, Teine-ku, Sapporo 006-8555, Hokkaido, Japan; y.koba1021@gmail.com (Y.K.); kohta294@yahoo.co.jp (Y.F.); wa_shin_2002@yahoo.co.jp (S.W.); 3 Mommy’s Clinic Chitose, 2-1-13 Shinano, Chitose 066-0038, Hokkaido, Japan; shimashige@hotmail.co.jp; 4 Department of Obstetrics and Gynecology, University of Yamanashi, 1110 Shimokato, Chuo 409-3898, Yamanashi, Japan; nyarometatsuyafanta@gmail.com (T.Y.); oyoshino624@gmail.com (O.Y.); 5 Department of Obstetrics and Gynecology, Hakodate Central General Hospital, Honchou 33-2, Hakodate 040-8585, Hokkaido, Japan; sorokata@hakochu-hp.gr.jp; 6 Center for Recurrent Pregnancy Loss, Teine Keijinkai Hospital, 1-40, 12-chome, Maeda, Teine-ku, Sapporo 006-8555, Hokkaido, Japan

**Keywords:** bacterial vaginosis, preterm birth, real-time PCR, *Ureaplasma*

## Abstract

Preterm birth is a major cause of perinatal mortality and morbidity in newborns, and its risk is increased by bacterial vaginosis (BV) during pregnancy. This multicenter prospective cohort study aimed to evaluate whether Flora select™ (FS), a newly developed real-time polymerase chain reaction test, is clinically useful for assessing the effectiveness of BV treatment during pregnancy. The vaginal microbiome characterized by relative dominance rates of *Lactobacillus* ≤ low (<50%), together with a positive test for *Gardnerella*, *Prevotella*, or *Atopobium* species, was defined as BV-FS A criterion. The vaginal microbiome characterized by *Lactobacillus* medium (50%≤, <80%), together with positive tests for *Gardnerella* plus either *Prevotella* species or *Atopobium* species, was defined as BV-FS B criterion. This study enrolled 25 pregnant women with classical BV (Nugent score ≥ 7) at initial examinations, and they met the BV-FS A (*n* = 23) and BV-FS B (*n* = 2) criteria. No woman with classical BV had a missed diagnosis of molecular BV. Treatments with metronidazole vaginal tablets resulted in the improvement of 88.0% (22/25) of classical BV, 65.2% (15/23) of BV-FS A, and 50.0% (1/2) of BV-FS B cases, whereas positive rates of *Ureaplasma* species in women with classical BV increased by 42.9%. Although most classical BV cases were cured following metronidazole treatments, a considerable proportion still harbored molecular BV detected by FS. Although the Nugent scoring system revealed that 80.0% (20/25) of women with classical BV (Nugent score ≥ 7) were sufficiently cured as BV-negative (Nugent scores 0–3), 5 (25%) of the 20 cured cases still met the BV-FS A/B criteria. FS particularly detected *Ureaplasma* species in 9 (45%) of the 20 cured cases. It could identify pregnant women who require additional treatments for residual molecular BV and *Ureaplasma* species. Therefore, the FS test may be clinically useful for assessing the vaginal microbiome and evaluating the effectiveness of BV treatments.

## 1. Introduction

Preterm birth is a major cause of perinatal mortality and morbidity in newborns, and its risk is increased by bacterial vaginosis (BV) during pregnancy [1]. The stabilization or colonization of several vaginal bacterial species, such as *Gardnerella* and *Bacteroides*, causes BV. Classical clinical signs and symptoms in Amsel’s criteria [2] or the microscopically based Nugent score [3] have been used for diagnosing BV over the years. However, the morphological assessment of bacterial species is time-consuming and somewhat subjective, and it cannot accurately identify the pathogens [4]. In addition, molecular epidemiologic studies have shown that *Ureaplasma* and *Mycoplasma* species are more frequently detected in women with BV as part of the vaginal dysbiosis [5]. The more consistent evidence links these microorganisms to adverse pregnancy outcomes, especially preterm birth [6].

Molecular testing using real-time polymerase chain reaction (PCR) and 16S rRNA sequencing for BV diagnosis has been introduced [4,7,8]. A randomized clinical trial on pregnant women revealed that real-time PCR tests and treatment for BV, based on the quantification of *Gardnerella* and *Atopobium*, significantly reduced preterm birth rates [9]. Recently, we reported a prospective cohort study evaluating the performance of Flora select™ (FS) (Varinos Inc., Tokyo, Japan), a newly developed real-time PCR test, for the assessment of the vaginal microbiome during early pregnancy [10]. In this study, 556 pregnant women underwent Nugent scoring for BV diagnosis, conventional bacterial culture, and FS tests. FS revealed that the proportion of *Lactobacillus* relative dominance rates of high (≥80%), medium (50% ≤ medium < 80%), low (0.1% ≤ low < 50%), and no detection (<0.1%) were 63.0%, 8.8%, 17.1%, and 11.2%, respectively. *Gardnerella*, *Prevotella*, *Atopobium*, *Streptococcus*, *Ureaplasma*, and *Mycoplasma* species were detected in 23.9%, 17.6%, 17.1%, 7.0%, 23.0%, and 4.9% of these women, respectively. Compared with conventional bacterial culture, FS detected *Gardnerella*, *Prevotella*, and *Atopobium* species more effectively. FS could determine relative dominance rates of *Lactobacillus* species in the vaginal microbiome, and simultaneously detect these BV-associated bacteria along with *Ureaplasma* and *Mycoplasma* species. Thus, the previous study demonstrated the clinical usefulness of vaginal microbiome screening during pregnancy to prevent preterm birth [10].

This multicenter prospective study aimed to evaluate whether FS, a real-time PCR test, is clinically useful for assessing the effectiveness of BV treatment during pregnancy.

## 2. Materials and Methods

### 2.1. Study Participants

This prospective cohort observational study was conducted between May 2024 and February 2025. It adhered to the guidelines of the Declaration of Helsinki and was approved by the Institutional Review Board of Teine Keijinkai Hospital (No. 2-023272-00, dated 17 November 2023). Written informed consent was obtained from all participants in four hospitals/clinics: Sapporo Toho Hospital, Mommy’s Clinic Chitose, Teine Keijinkai Hospital, and University of Yamanashi Hospital. Pregnant women underwent examinations of FS tests, together with Nugent scoring—a Gram-staining scoring system for BV diagnosis—between 8 and 12 weeks of gestation at regular maternity checkups. Two swabs were used to obtain vaginal fluid. The first swab was used for the Nugent scoring, and the second for FS.

Women diagnosed with classical BV based on a Nugent score ≥ 7 received a 250 mg metronidazole vaginal tablet once a day for 7 days. After approximately 4 weeks of treatments, they underwent FS tests and Nugent scoring to assess the treatment effectiveness. FS results and Nugent scores were compared to evaluate whether FS is clinically useful for assessing the vaginal microbiome after BV treatments.

### 2.2. Procedures

#### 2.2.1. Nugent Score

The Nugent scoring system evaluates bacterial morphotypes microscopically for BV diagnosis using the Bartholomew and Mittwer methods (Muto Pure Chemicals Co., Ltd., Tokyo, Japan) and the Favor method (Shimadzu Diagnostics Co., Tokyo, Japan) for Gram staining. Nugent scores vary from 0 to 10, based on the quantitative presence of three bacterial morphotypes, which are assessed in Gram-stained vaginal fluid. The quantitative number of *Lactbacillus* morphotypes is scored 0–4, where 0 indicates the lowest amount. Small Gram-variable rods (*Gardnerella vaginalis* and *Bacteroides* morphotypes) are scored 0–4, with 4 indicating the highest amount, and curved Gram-variable rods (*Mobiluncus* morphotypes) are scored 0–2, with 2 indicating the highest amount. BV is diagnosed when Nugent scores are ≥7 [3,10]. Nugent scores of 0–3, 4–6, and ≥7 indicate BV-negative, BV-intermediate, and BV-positive.

#### 2.2.2. Real-Time PCR Test (Flora Select™)

The samples for Nugent scoring, bacterial culture, and Flora select™ (FS, Varinos Inc., Tokyo, Japan) were simultaneously obtained by swabbing the vaginal walls using two different swabs. Vaginal-swab samples for FS were directly collected in a Copan eNAT collection tube (Copan Italia, Brescia, Italy). The samples were stored at room temperature until DNA extraction, which was performed within 4 weeks according to the manufacturer’s protocol. DNA extraction and the amplification of bacterial DNA by real-time PCR, using the SYBR Green Method (TOYOBO, Osaka, Japan), was conducted by Varinos Inc. The samples were treated with proteinase K of ≥600 U/mL (Kanto Kagagu Co., Inc., Tokyo, Japan) and a lysozyme solution of 1.5 mg/mL (Merck KGaA, Darmstadt, Germany) for cell lysis as pretreatment. Genomic DNA was extracted using MagNA Pure 24 system (Pathogen 1000 hp 3.2 software/protocol, Roche Diagnostics GmbH, Mannheim, Germany).

The relative absolute abundance of *Lactobacillus* species was calculated by the ratio of the amount of *Lactobacillus* to the total amount of bacterial species. The cycle threshold (Ct) of each sample was compared with that of the standard curve made by diluting the genomic DNA of *Lactobacillus crispatus.* The abundance of *Lactobacillus* was classified into four categories, based on relative dominance rates of *Lactobacillus* species, namely high (≥80%), medium (50%≤, <80%), low (0.1≤, <50%), and no detection (<0.1%). The four BV-associated bacterial species (*Gardnerella*, *Atopobium*, *Prevotella*, and *Streptococcus*) and two miscarriage/preterm birth-associated bacterial species (*Ureaplasma* and *Mycoplasma*) were detected by multiplex PCR using specific primers for each bacterium. Primers for the four BV-associated bacteria and *Ureaplasma* were designed to amplify all known species in each genus and targeted at the 16S or 23S rRNA gene region. Two sets of primers were designed to amplify *Mycoplasma* species, including *Mycoplasma genitalium* and *hominis*, which are found in female reproductive organs.

The threshold for a positive result for each bacterium was adjusted to the threshold of a conventional bacterial culture test. The amplification reactions were performed on a CFX96 C1000 Touch Real-Time System (CFX Maestro, Bio-Rad Laboratories, Inc., Hercules, CA, USA), using a total volume of 20 μL containing THUNDERBIRD Next SYBR qPCR Mix (TOYOBO Co., Ltd., Osaka, Japan), forward and reverse primer sets (at 0.5 μM concentration), and the extracted bacterial DNA. Each plate included a no-template control and a positive control. The correlation coefficient between FS and next-generation sequencing for relative dominant rates of *Lactobacillus* species was 0.995. The positive and negative predictive values of FS for known DNA samples of the six bacteria were found to be 100%.

When FS detected relative dominance rates of *Lactobacillus* ≤low (<50%), together with a positive test for *Gardnerella*, *Prevotella*, or *Atopobium* species, the vaginal microbiome was defined as the BV–Flora select™ (BV-FS) A criterion. The vaginal microbiome of *Lactobacillus* medium (50%≤, <80%), together with positive tests for *Gardnerella* plus either *Prevotella* species or *Atopobium* species, was defined as the BV-FS B criterion. Molecular BV was defined as meeting BV-FS A/B criteria, which can be detected by FS tests.

### 2.3. Statistical Analysis

Fisher’s exact tests were used for the comparison of improvement rates of classical BV, BV-FS A criterion, and BV-FS B criterion. Significance was set at *p* < 0.05.

## 3. Results

A total of 25 pregnant women diagnosed with BV based on a Nugent score ≥ 7 were enrolled and received 250 mg metronidazole vaginal tablets for 7 days. Table 1 shows the characteristics of participants and FS results at initial examinations. Relative dominance rates of *Lactobacillus* species of high (≥80%), medium (50%≤, <80%), low (0.1≤, <50%), and no detection (<0.1%) were 0%, 8%, 68%, and 24%, respectively. *Gardnerella*, *Prevotella*, *Atopobium*, and *Streptococcus* species (BV-associated bacteria) were detected in 96%, 24%, 84%, and 24% of the 25 women, respectively. *Ureaplasma* and *Mycoplasma* species (miscarriage/preterm birth-associated bacteria) were detected in 28% and 8% of the women, respectively.

All 25 women with classical BV (Nugent score ≥7) met the BV-FS (*n* = 23) and BV-FS B (*n* = 2) criteria. No woman with BV had a missed diagnosis of molecular BV. Table 2 shows changes in vaginal microbiome after treatments with metronidazole in 25 women with classical BV. The treatments reduced the number of women with classical BV, BV-FS A/B, and positive rates of *Mycoplasma.* Improvement rates of classical BV, BV-FS A, and BV-FS B were 88.0% (22/25), 65.2% (15/23), and 50.0% (1/2), respectively. However, the proportion of women who tested positive for *Ureaplasma* increased by 42.9%, from 7 to 10 women, after the treatments. The improvement rates of the BV-FS A/B criteria were lower than those of classical BV; however, the difference was not statistically significant.

Table 3 shows Nugent scores and vaginal microbiome after treatments in women with classical BV (Nugent score ≥ 7). After treatments, 20 women (80.0%) had Nugent scores 0–3 (BV-negative), 2 (8.0%) had 4–6 (BV-intermediate), and the remaining 3 (12.0%) had ≥7 (BV-positive). FS revealed that a significant proportion of women still had BV-FS A/B and tested positive for *Ureaplasma* species even after achieving Nugent scores 0–3 (BV-negative). Five (25.0%) of twenty women with Nugent scores 0–3 were diagnosed with BV-FS A/B, and nine (45.0%) tested positive for *Ureaplasma* species. One (50.0%) of two women with Nugent scores 4–6 (BV-intermediate) was diagnosed with BV-FS A. All three women with a Nugent score ≥ 7 were diagnosed with BV-FS A.

**Figure 1:**
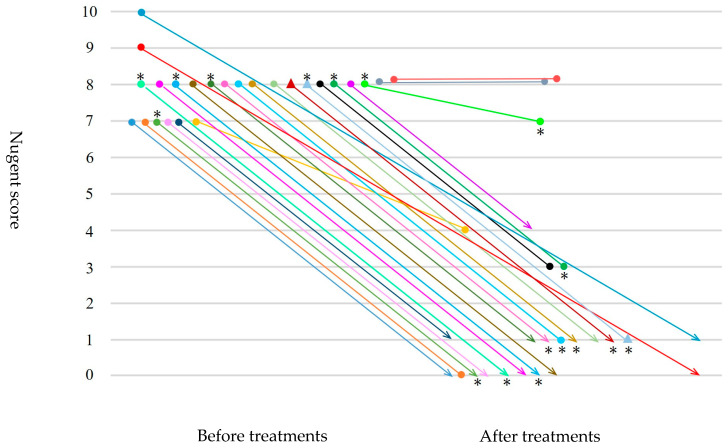
Changes in Nugent scores, BV-FS A/B criteria before and after treatments with metronidazole in 25 women with classical BV (Nugent score ≥7). ● Bacterial vaginosis–Flora select™ (BV-FS) A criterion. ▲ Bacterial vaginosis–Flora select™ (BV-FS) B criterion. ∗ Ureaplasma-positive.

Figure 1 shows changes in Nugent scores, and BV-FS A/B criteria before and after treatments in 25 women with classical BV (Nugent score ≥ 7). All eight women with BV-FS A and one woman with BV-FS B after treatments had been diagnosed with BV-FS A and BV-FS B at initial examinations, respectively. No changes in the status of BV-FS A/B conditions were observed before and after treatments. One of seven cases with positive tests for *Ureaplasma* species at initial examinations was cured after treatments, whereas four cases with negative tests for *Ureaplasma* species at initial examinations tested positive after treatments.

## 4. Discussion

This study evaluated the clinical usefulness of FS, a newly developed real-time PCR test, for assessing the vaginal microbiome after BV treatments. Of the 25 pregnant women with classical BV (Nugent score ≥ 7) at initial examinations, 23 satisfied the BV-FS A criterion and 2 met the BV-FS B criterion. No woman with classical BV had a missed diagnosis of molecular BV. Treatments with metronidazole vaginal tablets improved 88.0% (22/25) of classical BV, 65.2% (15/23) of BV-FS A, and 50.0% (1/2) of BV-FS B cases, whereas positive rates of *Ureaplasma* species in women with classical BV increased by 42.9%, from 7 to 10 women. Most cases with classical BV were cured after metronidazole treatments, while a considerable proportion still harbored molecular BV detected by FS. Although the Nugent scoring system revealed that 80.0% (20/25) of women with classical BV were sufficiently cured, being BV-negative (Nugent scores 0–3), 5 (25%) of the 20 cured women still met BV-FS A/B criteria. The FS test particularly detected *Ureaplasma* species, which cannot be detected by Gram staining, in 9 (45%) of the 20 cured women. Therefore, FS appeared clinically useful for assessing the effectiveness of treatments and for preventing preterm birth.

In a previous study, 121 (21.8%) of 556 pregnant women had at least one of BV-associated bacterial species (*Gardnerella*, *Prevotella*, *Atopobium*, or *Streptococcus*), together with no detection (<0.1%) or a low level of *Lactobacillus* (0.1≤, <50%), while 11 (2.0%) had *Gardnerella* plus one more BV-associated bacterial species, together with medium *Lactobacillus* (50%≤, <80%) [10]. In the present study, these microbiome states indicated molecular BV, BV-FS A and BV-FS B, respectively, in evaluating the effectiveness of BV treatments. This study revealed that BV-FS A/B criteria covered all women with classical BV at initial examinations, and they were more sensitive in assessing the vaginal microbiome after the treatments.

Moreover, FS could determine relative dominance rates of *Lactobacillus* species in the vaginal microbiome, and simultaneously detect four kinds of BV-associated bacteria species, namely *Gardnerella*, *Prevotella*, *Atopobium*, and *Streptococcus*, alongside *Ureaplasma* and *Mycoplasma* species, which cannot be detected by Gram staining or conventional bacterial culture [10]. *Ureaplasma* species are microorganisms frequently isolated from the amniotic fluid and placentae of cases with preterm delivery, and they are associated with miscarriages, neonatal respiratory diseases, and chorioamnionitis [11]. The presence of *Ureaplasma* species in the vaginal microbiome early in pregnancy was causally associated with subsequent miscarriages and preterm delivery [12,13]. The presence of *Ureaplasma* species in the uterine endometrium before pregnancy increased the risk of adverse pregnancy outcomes in women with recurrent pregnancy loss [14]. Therefore, FS tests demonstrated a definite advantage over the Nugent scoring system for assessing the vaginal microbiome in estimating preterm delivery risk and in evaluating the effectiveness of BV treatments. The combined use of the Nugent scoring system and FS will be clinically useful for preventing preterm birth, particularly in high-risk pregnancies, and facilitate the accurate identification of pregnant women who require additional treatments for residual molecular BV and *Ureaplasma* species.

This prospective study has several limitations. The number of participants was relatively small, only 25 women. All participants were Japanese; the human vaginal microbiome changes according to race, diet, living environment, and life-cycle stage. This study was limited by not being able to separate species, but only genera. Differences in *Lactobacillus* species (*L. crispatus*, *gasseri*, *jensenii*, and *iners*) were not assessed. Japanese national health insurance approves the maximum dosage of metronidazole vaginal tablet of 250 mg per day for 7 days for BV treatments in pregnant women, whereas CDC guidelines recommend a higher total dose (e.g., oral metronidazole 500 mg twice daily for 7 days or 0.75% metronidazole gel once daily for 5 days). BV is a complex imbalance of the vaginal microbiome. Naturally, the Nugent score does not identify all bacteria associated with BV, but based on morphotype count, it suggests the possibility of BV. Pregnancy outcomes (miscarriage, preterm birth, and term delivery) of participants in relation to FS results or Nugent scores were not evaluated. Thus, further prospective studies are needed to determine whether persistent detection of *Ureaplasma* or *Gardnerella* after treatments correlates with adverse obstetric outcomes. These should be clarified in further investigations.

## 5. Conclusions

This study evaluated the clinical usefulness of FS, a real-time PCR test, for assessing the vaginal microbiome after BV treatments. Treatments with metronidazole vaginal tablets improved 88.0% of classical BV, 65.2% of BV-FS A, and 50.0% of BV-FS B cases, whereas positive rates of *Ureaplasma* species in women with BV increased by 42.9%. Most classical BV cases were cured after metronidazole treatments, while a considerable proportion still harbored molecular BV detected by FS. The Nugent scoring system revealed that 80.0% of women with BV were sufficiently cured, being BV-negative; however, 25% of these women still met BV-FS A/B criteria. The FS test particularly detected *Ureaplasma* species, which cannot be detected by Gram staining, in 45% of the cured women.

FS tests may have an advantage over the Nugent scoring system. FS could identify pregnant women who require additional treatments for residual molecular BV and *Ureaplasma* species. Therefore, FS may be clinically useful for assessing the vaginal microbiome and for evaluating the effectiveness of BV treatments.

## Figures and Tables

**Table 1:** Characteristic of participants and results of Flora select™.

Characteristics of Participants *n* = 25	
Age, years	30 (23–38)
Body mass index, kg/m^2^	22.0 (16.0–32.9)
Nulliparous, %	13 (52%)
Number of previous miscarriages, %	0 (0–2)
Number of previous preterm birth	0
Assisted reproductive technology	0
Artificial insemination with husband’s sperm, %	1 (4%)
Gestational weeks at initial examination	9 (8–12)
Nugent score at initial examination	8 (7–10)
Gestational weeks at the second examination	16 (12–20)
Cervical length in mid-pregnancy, mm	41.3 (30.6–54.8)
**Flora select™ at Initial Examination**	
Relative dominance rate of *Lactobacillus* species	
High (≥80%)	0
Medium (50%≤, <80%)	2 (8%)
Low (0.1%≤, <50%)	17 (68%)
No detection (<0.1%)	6 (24%)
Presence of BV-associated bacteria species	
*Gardnerella*	24 (96%)
*Prevotella*	6 (24%)
*Atopobium*	21 (84%)
*Streptococcus*	6 (24%)
Miscarriage/preterm labor-associated bacteria species	
*Ureaplasma*	7 (28%)
*Mycoplasma*	2 (8%)
Median (range); BV, bacterial vaginosis	

**Table 2:** Changes in vaginal microbiome after treatment with metronidazole in pregnant women with bacterial vaginosis (Nugent score ≥ 7).

	Nugent Score ≥ 7	BV-FS A Criterion	BV-FS B Criterion	*Ureaplasma* Positive	*Mycoplasma* Positive
Initial examination	25	23	2	7	2
After treatment	3	8	1	10	0

BV-FS A criterion; *Lactobacillus* ≤low (<50%) and a positive test for *Gardnerella*, *Prevotella*, or *Atopobium* species. BV-FS B criterion; *Lactobacillus* medium (50%≤, <80%) and positive tests for *Gardnerella* plus either *Prevotella* species or *Atopobium* species.

**Table 3:** Vaginal microbiome after treatment with metronidazole according to Nugent scores.

Nugent Score After Treatment	Number	BV-FS A Criterion *n* = 8	BV-FS B Criterion *n* = 1	*Ureaplasma* Positive *n* = 10
0–3	20	4	1	9
4–6	2	1	0	0
≥7	3	3	0	1

BV-FS A criterion; *Lactobacillus* ≤low (<50%) and a positive test for *Gardnerella*, *Prevotella*, or *Atopobium* species. BV-FS B criterion; *Lactobacillus* medium (50%≤, <80%) and positive tests for *Gardnerella* plus either *Prevotella* species or *Atopobium* species.

## Data Availability

The data presented in this study are available on request from the corresponding author due to privacy reasons.

## Abbreviations

The following abbreviations are used in this manuscript:

BV	Bacterial vaginosis
FS	Flora select™
PCR	Polymerase chain reaction
